# Tooth Organ Bioengineering: Cell Sources and Innovative Approaches

**DOI:** 10.3390/dj4020018

**Published:** 2016-06-02

**Authors:** Hasan A. Jamal

**Affiliations:** Independent Researcher, Ibrahim Al- Jaffali, Awali, Mecca 21955, Saudi Arabia; hasan.jamal@kcl.ac.uk; Tel.: +966504519996

**Keywords:** dental stem cells, regeneration, repair, stem cell niche, craniofacial biology, stem cell biology, regenerative dentistry, tissue engineering

## Abstract

Various treatment approaches for restoring missing teeth are being utilized nowadays by using artificial dental crowns/bridges or the use of dental implants. All aforementioned restorative modalities are considered to be the conventional way of treating such cases. Although these artificial therapies are commonly used for tooth loss rehabilitation, they are still less conservative, show less biocompatibility and fail to restore the natural biological and physiological function. Adding to that, they are considered to be costly due to the risk of failure and they also require regular maintenance. Regenerative dentistry is currently considered a novel therapeutic concept with high potential for a complete recovery of the natural function and esthetics of teeth. Biological-cell based dental therapies would involve replacement of teeth by using stem cells that will ultimately grow a bioengineered tooth, thereby restoring both the biological and physiological functions of the natural tooth, and are considered to be the ultimate goal in regenerative dentistry. In this review, various stem cell-based therapeutic approaches for tooth organ bioengineering will be discussed.

## 1. Introduction

Tooth loss and the outbreak of oral diseases, such as tooth decay, periodontal disease, and traumatic injury, causes significant complications for oral function, including articulation, mastication, and occlusion, along with general health issues [1]. Currently, osseo-integrated dental implants are used to restore missing teeth. Although these artificial therapies are commonly utilized for tooth loss rehabilitation, they are still less conservative, show less biocompatibility, and fail to restore the natural biological and physiological functions. On the other hand, cell-based dental therapies would involve the replacement of teeth by using stem cells that will ultimately grow a bioengineered tooth, thereby restoring both the biological and physiological functions of the natural tooth, and are considered to be the ultimate goal in regenerative dentistry. This review will, firstly, give an overview of the cell sources used for whole tooth bioengineering. Secondly, the current approaches will be discussed. Finally, some of the biological constraints encountered during whole-tooth bioengineering construction will be addressed. Due to the extensity of the corresponding literature, a selection of significant publications will be presented.

### Tooth Organogenesis

During embryonic development, tooth organogenesis is organized by complementary epithelial and mesenchymal interactions; a principle developmental apparatus associated with stem cells, transcription factors, and signaling molecules. The dental lamina starts to thicken (lamina stage), followed by epithelial thickening (placode stage). In craniofacial development in mice, tooth- forming sites are specified on embryonic day (ED) 10–11 by the expression of certain genes, such as Msx1, Msx2, Lhx8, and Barx1, and secretory molecules, such as fibroblast growth factors (FGFs) and bone morphogenetic proteins (BMPs) [2,3]. These signals induce the expression of a number of transcription factors in the dental mesenchyme that is condensed around the developing epithelial bud (bud stage). During ED13.5–14.5, an enamel knot is formed, and it acts as a signaling center regulating tooth development by controlling gene expression (cap stage). At ED16–18, epithelial and mesenchymal cells differentiate into tooth tissue progenitor cells, including ameloblasts, odontoblasts, and dental follicle cells (bell stage). Ameloblasts form the enamel, odontoblasts form the dentine-pulp complex, while dental follicle cells differentiate into periodontal tissues (cementum, PDL, and alveolar bone).

A schematic frontal view of an embryo head at ED11.5 is shown with a dashed box to indicate the site where the lower (mandibular) molars will form. Below, the stages of tooth development are laid out from the first signs of thickening at ED11.5 to the eruption of the tooth at around five weeks after birth. The tooth germ is formed from the oral epithelium and neural-crest-derived mesenchyme. At the bell stage of development, the ameloblasts and odontoblasts form in adjacent layers at the site of interaction between the epithelium and mesenchyme. These layers produce the enamel and dentin of the fully formed tooth [2].

## 2. Sources of Whole Tooth Regeneration

### 2.1. Combination of Epithelium and Mesenchyme-Derived from Embryonic Tooth Germ Cells

Various studies concerning designing methods for bioengineering a whole tooth have been achieved by utilizing embryonic mouse tooth germ cells, as these cells allow full tooth development by cell-cell re-association [4,5]. The primary motive behind the use of mouse embryonic tooth germ cells is, firstly, the fact that a considerable amount of data are present regarding tooth development, and, secondly, due to the feasibility and convenience of studying mouse models in relation to the epithelial-mesenchymal interactions [6,7]. Lastly, the duration of organogenesis is quick enough for the experiments to be accomplished in a plausible period [8].

A morphogenetic center controlling crown morphogenesis (*i.e.*, Primary enamel knot, PEK) gradually forms in the enamel organ. Thus, it is fundamental to achieve both histogenesis and functionalization of this structure in cell-cell re-association. In a study, the primary enamel knot (PEK) was formed during initial stages of the re-association *in vitro* using embryonic dental cells and was represented by the expression of Sonic hedgehog (Shh), the condensation of non-dividing cells in the epithelium and the regional concentration of apoptosis [4]. After the characterization of the (PEK) in four days, cusps formed, asserting that this structure was indeed functional. Nonetheless, the possibility of the embryonic dental mesenchyme to initiate tooth formation was lost *in vitro* [9]. Experiments were made to rescue it by pre-culturing these cells in the presence of FGF2 [9,10]. However, neither FGF2 nor FGF1 and FGF8 in combination allowed tooth formation. To generate a whole tooth using embryonic dental cells, a two-step approach is followed; culturing re-associated single epithelial and mesenchymal cells for a while before implanting them [11,12]. At the time of the first step *in vitro*, crown formation began, and the mesenchymal cells specified both the inner and the outer dental epithelium. By the eighth day *in vitro*, epithelial histogenesis was fully achieved; cusp morphogenesis was initiated and functional differentiation of odontoblasts started. Cultured re-associated cells were then implanted under adult mice skin, and the construct was vascularized and dentine and enamel were mineralized [13].

It is still unknown whether human embryonic dental tissues acquire the same inductive potential as mouse tooth germs. However, a recent study showed impressive results addressing this issue. It was discovered that human dental epithelium from the cap stage, instead of the bell stage, was capable of inducing tooth formation when combined with human embryonic lip mesenchyme. On the contrary, human dental mesenchyme from the bell stage, instead of the cap stage, could induce both mouse embryonic second-arch epithelium and human keratinocyte stem cells to develop into enamel-secreting ameloblasts. Neither post-natal dental pulp stem cells (DPSCs) nor stem cells from human exfoliated deciduous teeth (SHED) were shown to have odontogenic potential [14]. These results demonstrate the conservation of odontogenic potential in both mouse and human dental tissues during early tooth development.

The main concerns in tooth organ engineering now remain in discovering adequate cells to be utilized, bearing in mind the specifics of tooth development [15,16]. Certainly, odontogenesis results from sequential and reciprocal interactions between both the epithelial cells and the ectomesenchymal cells, with a shift of the inductive potential from the epithelial cells to the ectomesenchymal cells. Consequently, mimicking this developmental process at the molecular context still needs to be investigated more when dealing with the current applicable cells [9]. For instance, failure of re-associations to develop into a tooth can be easily detected [11]. Nevertheless, the molecular markers that are required, and which would allow for an earlier detection of this information, are still unknown.

Although the use of embryonic cells is considered to be an excellent candidate to set up methodologies for tooth bioengineering, their utilization in the clinical context is arguable. They hold numerous drawbacks such as the possibility of teratoma formation when transplanted, immunological rejection by the host, and ethical concerns regarding their use. A more feasible alternative would be the use of stem cells of other adult organs

### 2.2. Combination of Epithelium and Mesenchyme Derived from Non-Embryonic Tooth Germ Cells

Odontogenesis is sustained by an interaction between both epithelial and mesenchymal cells, and the inductive potential passed from the epithelium to the ectomesenchyme at ED12–12.5 in mouse embryos [17]. Since the instructive potential is controlled, competent cells can replace responding ones. A number of studies have tested this strategy by cells from different sources, mainly to replace the epithelial cells. In regard to using non-dental cells to replace the mesenchyme, few studies have been conducted.

Bone marrow stem cells (BMSCs) can be harvested in a plentiful amount at all ages and exhibit similar characteristics as dental pulp stem cells (DPSCs), which will be elaborated later. They both form calcified-deposits *in vitro* [6], and regarding their gene expression, studies showed that both BMSCs and DPSCs have similar gene expression levels, with slight differences [18]. BMSCs were recombined with oral epithelial cells from an ED10 mouse embryo when it was still able to instruct the mesenchymal cells. BMDSCs were shown to respond to the epithelial signals and thereby were capable of forming tooth-like structures and soft tissues along with associated bony tissues [19]. In about 10% of the cases, bioengineered teeth were obtained after implantation in kidney capsules [19]. Moreover, recent studies have demonstrated that BMSCs can give rise to ameloblast-like cells since ameloblast cells are lost during human tooth eruption [11].

Another interesting alternative cell type are induced pluripotent stem cells (iPSCs). They can differentiate into any of the three germ layer cell types, being generated by introducing a number of factors, these being c-Myc, Klf4, Oct4, and Sox2, into somatic cells [20]. The reprogrammed iPSCS exhibited indistinguishable characteristics from those of human embryonic stem (ES) cells in cultures, and they expressed ES markers (SSEA-4, TRA-1-60, TRA-1-80, TRA-2-49, Nanog, Oct4, and Sox2). In contrast with embryonic stem cells, iPS cells represent a promising population and were considered to be a better alternative. They hold no ethical or political implications regarding their usage and they show no sign of immune rejection due to the fact that they are autologous cells (patient-specific cells). In addition, they are considered to be an unlimited source of cells and can be easily translated to clinical application in comparison to other types of cells. Previous studies demonstrated that mouse induced pluripotent stem (iPS) cells can differentiate into dental mesenchymal cells along with odontoblasts progenitor cells. In a recent study, neural crest-like cells (NCLCs) were derived from mice iPS cells. They were then recombined with the apical end of incisor dental epithelium and cultured for two weeks, and by using molecular markers, it was concluded that these neural crest-like cells could be induced into odontoblast-like differentiation [21]. Complementary results, in which tooth-like structures with newly formed bone-like and dental-pulp-like tissues were generated, were obtained by mixing iPSCs with incisor mesenchymal cells [22]. A more recent study focused on ameloblastic differentiation capability of integration-free human urine-derived iPS cells (ifhU-iPSCs). These cells were initially differentiated into epithelial cells, followed by recombination of induced epithelial sheets with ED14.5 mouse molar mesenchyme. After three weeks in sub-renal culture, intact tooth- like structures were formed [23]. Not long after this study, another group showed that iPSCs can differentiate into ameloblast-like cells in cultures using feeder cells. They then followed that by another study in which they induced the differentiation of ameloblast-like cells from iPSCS under feeder-free conditions using medium conditioned by cultured epithelial cell rests of Malassez (ERM) cells and gelatin-coated dishes. By using the conditioned medium, they found greater evidence of ameloblast-like cell differentiation in the cultures. The expression of both amelogenin and ameloblastin were found to be significantly higher compared to controls [24].

In a recent study, adult human gingival epithelial cells were used to bioengineer a whole-tooth. Cells were isolated from adult human gingival epithelium and expanded *in vitro*, and were combined with embryonic mesenchymal cells of a mouse tooth. After seven days of culture, the re- associated cells were transplanted into kidney capsules of immunocompromised adult mice.

The result was a bioengineered tooth containing dentine, enamel with ameloblast-like cells, and the rest of malassez of human origin [25]. In another study, keratinocytes isolated from human foreskin appeared to be able to differentiate into enamel-secreting ameloblasts when recombined with embryonic mouse mesenchyme holding an odontogenic potential [26]. These findings highly contribute to having a sufficient cell population, as these cells are easy to access and to harvest.

In 2015, a study used human umbilical cord mesenchymal stem cells (hUCMSCs) to generate a whole-tooth. The author reported that hUCMSCs can be induced into odontoblast-like cell both *in vitro* and *in vivo*, and, with molecular markers, the cells expressed dentine-related proteins including dentine sialoprotein (DSP) and dentine matrix protein-1 (DMP-1), and their gene expression resembled those of native pulp tissue cells. This study put forward an alternative cell population as a therapeutic approach in bioengineering a whole tooth [27].

## 3. Sources of Partial Tooth Regeneration

### Root Regeneration

Since achieving accurate crown shape and size remain under investigation, it has been suggested that attempts should be shifted and centered toward the engineering of a functional anchorage apparatus (root, periodontal ligaments, and alveolar bone) on which an artificial crown might then be placed [15,28].

The root is considered to be the most crucial part of the tooth, since it acts as an anchorage for the whole tooth apparatus and it mediates tooth movements in the jaw. Root development in cell-cell re-association has been addressed in a number of studies [11,13,29]. Root formation could be initiated using dental embryonic cells only after the implantation of cell-cell re-associations cultures [11]. In these implants, periodontium similar to periodontal ligament fibers was formed [11]. During the two weeks of implantation, the periodontium continued being a non-mineralized zone in-between the dentine and cementum and new bone was formed [13]. Further periods of implantation need to be examined to ascertain whether the lack of mineralization of this region could be preserved. The primary cells in this process are Hertwig’s epithelial root sheath (HERS) cells [30]. They are found to be associated with the initiation process of the root formation and involved in stimulating cementogenesis and dentinogenesis [31]. Accordingly, for tooth engineering to take place, active HERS cells differentiation and maintenance are required.

During root formation, physiological cell heterogeneity in the periodontal mesenchyme should be reproduced, including its vascularization and innervation [32,33]. In this regard, work should be focused towards designing a system where the early stages of odontogenesis may greatly encourage the process, due to the progressive specification of the periodontal mesenchyme during development [34]. This aim was feasible via using dental embryonic cells. However it remains a major issue when using dental or non-dental stem cells. An experiment was conducted by co-culturing rat dental follicle cells (DFCs) and dental pulp cells (DPCs) on both sides of a polycarbonate membrane (0.4-micron pore size), thereby demonstrating that DPCs could influence the differentiation of DFCs to form periodontal components [35]. The experiment showed that DFCs solely formed fibrous tissue, however, when committed to osteogenesis, they managed to maintain these particular characteristics. This also showed that the presence of the polycarbonate membrane perturbed direct contact between these cell populations.

Commonly, most of the previous studies on human origin cells were achieved using stem cells from either the dental mesenchyme—dental pulp stem cells (DPSCs), stem cells from human exfoliated deciduous teeth (SHED), stem cells from the root apical papilla (SCAP), and stem cells from the dental follicle (DFSCs)—or the peridental mesenchyme and periodontal ligament stem cells (PDLSCs). The capabilities of these cells in dental tissue repair were tested. Gronthos *et al.* started a pioneering work and demonstrated that DPSCs could be induced to form a dentine/pulp-like complex [6]. Equivalently, SHED cells were demonstrated to have the capacity to induce bone formation, generate dentine, and differentiate into different non-dental mesenchymal cell derivatives [26]. DFCSs were found to have the ability to form cementoblast-like cells [36,37]. PDLSCs, on the other hand, were found to produce cementoblast-like cells and cementum/periodontal-like tissues [38,39].

Nevertheless, the use of these cells in bioengineering a whole tooth is rather vague, which is indeed related to the absence of epithelial cells that are able to induce mesenchymal stem cells to start an odontogenesis. In this context, there is no approach to instruct epithelial stem cells to act as early inductive cells [15].

## 4. Bioengineering Approaches for Whole Tooth Organ Regeneration

There are mainly two different approaches in the literature to bioengineering a tooth organ. The first approach is accomplished by associating dental epithelial stem cells (DESCs) and dental mesenchymal stem cells (DMSCs) *in vitro*, where they form a tooth primordium (explant) that could then be implanted into the alveolar bone to develop further and erupt as a functional tooth [19,40,41]. The second approach is made by implanting to the alveolar bone a prefabricated degradable scaffold that resembles tooth morphology seeded with both DESCs and DMSCs that will eventually give rise to a functional tooth [42].

### 4.1. A Novel 3D-Organ Germ Culture Method

In order to generate a three-dimensional ectodermal organ, such as salivary glands, hair follicles or even teeth, organogenesis has to be replicated, and this is achieved by simulating the epithelial-mesenchymal interaction that occurs during organ development [43]. When this replication is accomplished in a proper manner, a fully functional bioengineered organ will be generated. In regard to tooth regeneration, a proof of concept was put forward in which a bioengineered tooth primordium was transplanted into mouse alveolar bone and, as a result, a mature functional tooth developed [40]. It was suggested that mature bioengineered organ transplantation would provide an immediate full function *in vivo* [44]. The main challenge to produce a whole tooth explant is to establish a three-dimensional cell manipulation technology by using completely dissociated epithelial and mesenchymal cells *in vitro*. To date, two conventional approaches and a novel three-dimensional cell manipulation method for generating a bioengineered tooth primordium or a mature tooth have been investigated and will be described further in the review.

To achieve precise replication of organogenesis, an *in vitro* three-dimensional novel cell manipulation method was designed as the bioengineered organ germ method had already been developed [5]. This novel technique was established via compartmentalization of epithelial and mesenchymal cells at a high cell density in a collagen gel (hydrogels) *in vitro*. This method simulated the multicellular assembly of both epithelial-mesenchymal interactions in bioengineering a tooth germ and could aid in reconstructing large-scale organs. It also regulated the crown width by controlling the contact area between epithelial and mesenchymal cell layers [45]. Moreover, a structurally correct tooth was formed when the bioengineered germ was constructed by this method both *in vivo* and *in vitro* [5]. Another study that utilized this approach demonstrated that not only an accurate tooth structure, but also a fully functional tooth, can be developed [41]; this will be discussed in further detail later in the review. A recent experiment also used the three-dimensional (3D) tissue engineering method to characterize intact postnatal dental tissue recombinant constructs, which resulted in forming a morphologically correct calcified crown consisting of both enamel and dentine [46,47].

### 4.2. Scaffolds as a Three-Dimensional Tooth Bioengineering Approach

Scaffolds are an innovative approach in bioengineering technology, with their main objective being to form a three-dimensional housing in a measured morphology for the desired cells to be seeded.

There are a number of scaffold biomaterials available in the market such as natural scaffold material, synthetic polymers, nanofiber self-assembling scaffolds, bio-ceramics, and hydrogels. In regenerative therapy, scaffolds were used clinically and showed a capability to generate tissues with analogous cellular formation in both bone and cartilage [48,49]. In regard to tooth regeneration, a number of studies used poly-l-lactate-co-glycolide copolymer and polyglycolic acid (PLA/PLGA) or collagen/gelatine sponge as scaffold materials, which were seeded with both epithelial and mesenchymal cells isolated from murine or sus tooth buds [42,50]. These studies reported that the teeth that regenerated were of similar shape and composition compared to natural teeth, but periodontal tissues were not observed [51,52].

Although scaffolds are beneficial in acting as cell growth-guides, suggesting great results in tissue engineering, they still have some drawbacks. Firstly, they act as a hurdle to the epithelial-mesenchymal interaction and to preserving a gradual cell differentiation. Secondly, some scaffolds such as collagen or PLGA scaffolds may delay the immune response mechanism. Lastly, they show vascularization deficiency when used *in vivo*. Since the maximum diffusion path for nutrients is 200–300 μm, nutrient supply for cells within a 3D-construct would be rather insufficient [53,54]. In this context, the construct utilized *in vitro* has mainly been implanted into animal models, however, even in animals, there is no ideal strategy to vascularize a large construct to be incorporated into the recipient vascular system. Possible methods to solve this dilemma is by using growth factors or modified chemical geometry of the scaffolds represent two possible methods to solve this dilemma [55]. Laschke *et al.* approached this issue by pre-vascularizing scaffolds by initially implanting PLGA-scaffolds into mice latterly and then inserting it into skinfold chambers. Skinfold chambers are ideal for observing the recipient tissue reaction to implanted tissue or materials via a glass window. In this experiment Laschke *et al.* observed blood circulation, vascularization angiogenesis, and inosculation, which were analyzed later by histology and immunohistochemistry, and found improvement in perfusion [56].

## 5. Biological Responses of Regenerated Teeth

The ideal goal of regenerative therapies is to develop fully functional bioengineered organs or tissues, that are capable of replacing damaged or lost organs following trauma, disease, or simply the effects of aging [40,48,57,58,59]. Various recent accomplishments in organ bioengineering therapy have put forward a legitimate proof of concept in regard to the application of bioengineered organs, as well as regenerative methods to be used [60]. Some of these therapies were clinically applied for myocardial infarction [61], corneal debilitation [62], and hepatic failure [63] via myocardial cells, oral mucosal-epithelial cells, and liver cells, accordingly. Most of these bioengineering achievements were accomplished by developing three-dimensional bioengineered tissues that consist of a single cell type using appropriate cell aggregation methods [5], or uniform cell sheets [64] and biodegradable materials [65]. For an ideal tooth replacement bioengineering therapy, the tooth developed from a bioengineered germ or from a transplanted bioengineered mature tooth unit must be capable of being well-integrated with the recipient edentulous bone region. They also must serve as a fully-functional organ, which includes sufficient mastication ability, biological collaboration with the periodontal ligament tissues, as well as the ability to respond to noxious stimulations in the maxillofacial compartment [1,66].

### 5.1. Integration with Periodontal Ligament Tissues

The natural eruption of teeth controlled by an autonomic mechanism involves dental germ cell compartments along with the surrounding alveolar bone [67,68]. During tooth development, dental follicular-cells encompass the tooth germ surface to give rise to cementum, periodontal ligament, and alveolar bone, which will eventually perturb the resorption of the alveolar bone covering the tooth germ during tooth eruption [67,68]. One of the obvious concerns in dental regenerative therapy is whether transplanting a bioengineered tooth germ primordium into the patient’s edentulous area will successfully erupt and appropriately occlude with the opposing teeth.

Transplantation of a bioengineered mature organ would be a superior approach since it would establish an immediate functional performance *in vivo* [69]. A bioengineered mature tooth unit was transplanted into a drilled bony-defect in murine jaw, and was successfully engrafted within 40 days, and the periodontal ligament of the implanted bioengineered tooth unit was preserved by successful bone integration [41]. The Knoop hardness test was utilized to measure enamel and dentine hardness to be compared to that of the natural tooth [40,41]. The analysis of the bioengineered tooth was found to be similar to those of the natural teeth. The previously mentioned cell-based therapeutic approaches propose the potential to successfully restore the masticatory function.

### 5.2. Responses to Mechanical Load

One of the main areas to be evaluated is the biological response of the bioengineered tooth towards mechanical stresses and loads. Biological oral functions require a continuous assistance between tooth apparatus and maxillofacial region through the connection of periodontal ligament [66]. Malfunctions to the periodontal ligaments in the case of periodontal diseases or periodontal injuries, lead to critical physiological problems both to functional mastication and to general health [1]. In addition, the periodontal ligament is a sophisticated structure that, as previously mentioned, helps in absorbing masticatory loads and maintaining orthodontic tooth movements [66,70,71]. To date, Ikeda *et al.* and Oshima *et al.* have shown that the periodontal ligaments of the bioengineered tooth have successfully achieved functional tooth movement, as well as an adequate bone remodeling [40,41]. These findings suggested that a bioengineered tooth could properly regenerate and be adapted to the mechanical forces comparable to that of a natural tooth. By contrast, this approach will be superior to that of the traditional dental implant since the dental implants lack PDL apparatus; thus they will lack adequate response to mechanical stresses that will eventfully propose failure to the whole implant. Since the periodontal ligament is considered to be an important component for an implant to be restored, the neural responses need to also be considered.

### 5.3. Proprioception Potential of Neuronal Responses of Regenerated Teeth (Tooth Innervation)

The trigeminal nerve and the sympathetic nerves are the nerves that supply teeth, and both the physiological function of the teeth and perception of noxious stimuli are regulated by the afferent nervous system in the maxillofacial region [72]. In the case of implants, the ability to sense this noxious stimulus is absent, due to the lack of innervation in the periodontal tissue [73,74]. One of the goals of bioengineering teeth is to restore innervation; however, it remains a challenge. In order to restore the neuronal response in a bioengineered tooth, many studies suggested that re-associated cell-cell implant must be correctly positioned in the jaw to be closer to the supplying nerves. However, it was shown to be rather impossible and difficult to control, since mice jaws are relatively small [75,76]. In the same motive, a preliminary study was performed to examine whether innervation of cultured cell-cell re-association could be obtained after implantation under the skin. For this project, embryonic dental cell-cell re-association was implanted along with dorsal root ganglia (DRG). After two weeks of implantation, immunostaining for the peripherin showed an axonal growth in the peridental tissue site, but not in the mesenchyme. The reason why the innervation of the dental mesenchyme failed could be due to the embryonic stage (ED14) that was used to form dental cells. Surely, at ED14, innervation remains constrained in physiological conditions and only becomes possible postnatally [77]. However, when these cells were re-associated with trigeminal ganglia in immunocompromised nude mice instead of ICR mice, the innervation of the dental mesenchyme was evident after 14 days.

The main issue in innervating a bioengineered tooth is to determine whether the main cause of innervation might be from the alveolar nerve when the bioengineered tooth is implanted. In this regard, interesting findings demonstrated that the neural regeneration, the re-entry of nerve fibers into the tooth, could be accomplished in bioengineered teeth. The bioengineered tooth was shown to exhibit a sufficient perceptive potential for nociceptive pain stimulus, and that included both pulp injury and endodontic treatment, and could transport these pain thresholds to the central nervous system through c-Fos immune-reactive neurons [40,41]. Although the previously mentioned studies demonstrated that the bioengineered tooth could indeed restore the neuronal responses and could be comparable to that of the natural teeth, further studies need to be done to further evaluate the issue.

## 6. Prospects and Concluding Remarks

One of the main hurdles to be considered before translating the bioengineered tooth replacement approach to clinics is to identify suitable cell sources. Potential human cell sources have been suggested, while autologous cell source banking is being established. The biological potentials of these cell sources have to be tested both *in vivo* and *in vitro*. The next challenge is to bioengineer large teeth, where optimal vascularization and innervation is crucial. An important issue for identifying a future tooth regenerative cell source is the identification of specific combinations of factors capable of reprogramming non-dental cells to dental epithelium and mesenchyme. Tooth regenerative therapy is now regarded as a fundamental study model for future replacement regenerative therapies that can be applied to more complex organs, and will contribute substantially to the knowledge and technology required to regenerate organs. All in all, while many problems are waiting to be solved, fast progress in the molecular study of tooth biology and development, thanks to new high-throughput technologies, will facilitate the realization of implantable bioengineered teeth.
